# Poor Dietary Quality and Patterns Are Associated with Higher Perceived Stress among Women of Reproductive Age in the UK

**DOI:** 10.3390/nu13082588

**Published:** 2021-07-28

**Authors:** Karim Khaled, Vanora Hundley, Fotini Tsofliou

**Affiliations:** 1 Department of Rehabilitation & Sport Sciences, Faculty of Health & Social Sciences, Bournemouth University, Bournemouth BH8 8GP, UK; ftsofliou@bournemouth.ac.uk; 2 Centre for Midwifery, Maternal & Perinatal Health, Faculty of Health & Social Sciences, Bournemouth University, Bournemouth BH8 8GP, UK; vhundley@bournemouth.ac.uk

**Keywords:** perceived stress, psychological, stress, diet quality, dietary patterns, women, reproductive age, childbearing age, a-priori, a-posteriori

## Abstract

The aim of this study was to investigate the association between stress and diet quality/patterns among women of reproductive age in UK. In total, 244 reproductive aged women participated in an online survey consisting of the European Prospective into Cancer and Nutrition food frequency questionnaire in addition to stress, depression, physical-activity, adiposity, and socioeconomic questions. An a-priori diet quality index was derived by assessing the adherence to Alternate Mediterranean Diet (aMD). A-posteriori dietary-patterns (DPs) were explored through factor analysis. Regression models were used to assess the predictors of the DPs. Participants mainly had medium (*n* = 113) aMD adherence. Higher stress levels were reported by participants with low aMD adherence. Participants with high aMD adherence were of normal BMI. Factor analysis revealed three DPs: fats and oils, sugars, snacks, alcoholic-beverages, red/processed meat, and cereals (DP-1), fish and seafood, eggs, milk and milk-products (DP-2), and fruits, vegetables, nuts and seeds (DP-3). Regression models showed that DP-1 was positively associated with stress (*p* = 0.005) and negatively with age (*p* = 0.004) and smoking (*p* = 0.005). DP-2 was negatively associated with maternal educational-level (*p* = 0.01) while DP-3 was negatively associated with stress (*p* < 0.001), BMI (*p* = 0.001), and white ethnicity (*p* = 0.01). Stress was negatively associated with healthy diet quality/patterns among reproductive aged women.

## 1. Introduction

Increased body weight before pregnancy is associated with higher risk of pregnancy complications [1,2,3,4] and of severe maternal morbidity and mortality [5]. A recent meta-analysis has indicated that a healthy diet is crucial to prevent increased weight gain before and during pregnancy and its related complications (e.g., gestational diabetes, preeclampsia, caesarean section delivery) [6].

There are several predictors of diet quality, one of which is perceived stress. Stress is increasing among people and has been associated with poorer diet quality among women of reproductive age [7,8,9,10,11]. However, most studies have focused on the association between stress and individual foods/food groups. For example, a higher level of psychological stress among women of reproductive age was found to be significantly associated with a greater consumption of fat in their diet [12,13,14,15]. Studies have also found that stress has been associated with decreased intake of fruits and vegetables among women of reproductive age [16,17]. Moreover, higher levels of perceived stress have been found to associate with increased consumption of sweets, fast foods, snacks, and saturated fats and decreased intake of fruits, vegetables, and unsaturated oils [18,19]. However, these studies assessed dietary intake through recall questionnaires that do not include a variety and wide range of food items and food groups; this might predispose participants to misreporting [8,20,21,22,23,24,25]. With respect to the evaluation of diet quality, most studies on stress and diet have included only the a-priori dietary approach (based on measuring adherence to diet index) [12,22,25]. To the best of our knowledge, no study on stress and diet have combined both the a-priori and a-posteriori (based on statistical techniques such as factor analysis) approaches which offer comprehensive insight and characterisation of the diet pattern specific to the population group under investigation. Additionally, the small sample sizes and the lack of representativeness in those studies mean that generalising the results is not possible. Not considering confounding factors such as sociodemographic characteristics and physical activity was also a major limitation of some studies [12,16,24,26,27]. Moreover, most studies on the association between stress and diet were conducted among the general adult population, however evidence is scarce among women of reproductive age (18–49 years old) [26,28,29]. 

In summary, studies on stress and diet in the literature have several limitations. Investigating the factors that affect diet quality in women of reproductive age has crucial importance especially because diet-related morbidity among these women had an increasing trend during the past years [30]. To our knowledge, this study is the first to examine the association between stress and diet quality among women of reproductive age in UK. The aim of this study is to investigate whether higher level of perceived stress is associated with lower diet quality among women of reproductive age in the UK. 

## 2. Materials and Methods

This was a cross sectional study targeting women of reproductive age in the UK. The study used an online questionnaire survey developed and administered via the Bristol Online Surveys (BOS). 

The sample was one of convenience and consisted of females of reproductive age (between 18 and 49 years old) who were students and staff at a UK University. There are varying definitions for reproductive age in the literature; the range for this study was chosen to reflect the majority of recent studies [31,32]. Participants were excluded if they were: males at birth, below 18 or above 49 years old, not students or staff, suffering from a chronic illness or disease such as: cancer, Crohn’s disease, diabetes, heart disease, HIV/AIDS/multiple sclerosis, depression, asthma, COPD, cystic fibrosis, or mental health disorder, having any kind of food intolerance or food allergy, pregnant or breastfeeding, or were on any medication known to affect appetite or body weight or have undergone bariatric surgery. The sample size was calculated by applying the correlation sample-size method [33] with a power of 80%, and an α (significance level) of 0.05. A correlation coefficient of 0.18 was chosen for the power calculation and it was based on the lowest correlation coefficient r reported in studies about stress and diet quality in women of reproductive age [24,30]. This yielded a total sample of 240 participants. 

Potential participants were targeted through posters and social media advertisements (e.g., twitter). Consent was ascertained on the landing page of the survey.

### 2.1. Methodological Measurements and Procedures

#### 2.1.1. Diet

Diet quality and patterns were estimated via the European Prospective into Cancer and Nutrition food frequency questionnaire (EPIC FFQ) which has been previously validated among UK adult females [34,35]. The EPIC-FFQ consists of 130 food items and one additional question for milk (131 items). The questionnaire represents either individual food (51%), combination of between two and four individual foods (23%), or food types (26%) that are further described by examples of individual foods. The number and percentage of food types in the list are: vegetables, 25 (19%); fruit and fruit juices, 12 (9%); meats, poultry, fish and eggs, 18 (14%); breads, cereals and starches, 18 (14%); dairy foods and fats, 15 (11%); beverages, 10 (8%); sweets and confectionery items, 14 (11%) and miscellaneous foods, 19 (14%). The food list is associated with a set of nine frequency choices for consumption ranging from ‘never or less than once a month’ to ‘6 or more times per day’. The questionnaire consists of two parts. Part 1, the main part, lists 130 food items. Part 2 includes a set of additional questions that determine further information on the type and brand of breakfast cereal and kind of fat used in frying, roasting, grilling or baking and the amount of visible fat on meat.

#### 2.1.2. Dietary Data Analysis

The FETA software was used to analyse the EPIC FFQ data and calculate the grams/day of nutrients and food groups [36]. Eleven food groups (grams/day) were derived from the EPIC food frequency questionnaire data analysis which included fats and oils, sugars and snacks, cereals, alcoholic beverages, red and processed meat, fish and seafood, eggs, milk and milk products, fruits, vegetables, and nuts and seeds. Adherence to the Alternate Mediterranean Diet Index (aMED) was used to assess the a-priori approach for diet quality assessment. The aMED is an adjustment of the Mediterranean Diet Index, which is based on the evidence suggesting a protective effect of this diet on the risk of chronic diseases, and that was previously developed by Trichopoulou et al. [37]. The aMED is based on the consumption of nine food groups: vegetables (excluding potatoes), fruit, nuts, legumes, fish, whole grains, mono-unsaturated fatty acids to saturated fatty acids ratio, alcohol, and red and processed meat [38]. The a-posteriori approach was based on factor analysis that derived the dietary patterns of participants. The importance of factor analysis (a-posteriori approach) is that it characterises the sample’s variation in dietary intake and provides a more meaningful description of the overall patterns and quality of the diet which complements the a-priori dietary approach [39]. 

#### 2.1.3. Mental Health Indicators

Perceived stress was measured using the 14-item Perceived Stress Scale (PSS) [40]. PSS measures the level of psychological stress, thoughts, and feelings of each participant over the past month. The scale has been tested in several trials in adult populations and showed significant consistency with Cronbach’s alpha = 0.75 and 0.85 [40]. The PSS is not a diagnostic tool; hence there are no cut-off points that determine if an individual is stressed. PSS was equally divided into two categories: low to medium level of stress (score = 0–27) and medium to high level of stress (score = 28–56) as per previous studies [11,41].

Depression was measured using the 21-item Beck Depression Inventory II (BDI-II) [42]. The BDI-II has become one of the most widely used measures to assess depressive symptoms and their severity in adolescents and adults [43]. The BDI-II is a 21-item self-report measure that taps major depression symptoms according to diagnostic criteria listed in the Diagnostic and Statistical Manual for Mental Disorders [44]. Since its publication, a number of studies have examined the validity and reliability of BDI-II across different populations and countries [45]. Results have consistently shown good internal consistency and test-retest reliability of the BDI-II in community [46,47] adolescent and adult clinical outpatients [48] as well as in adult clinical inpatients [49]. 

#### 2.1.4. Physical and Socio-Demographic Characteristics

Physical activity was measured using the International Physical Activity Questionnaire (IPAQ) [50]. The IPAQ records the activity of participants of four intensity levels: vigorous-intensity activity such as aerobic, moderate-intensity activity such as leisure cycling, walking, and sitting [50]. 

Adiposity measures: weight in kg and height in cm were self-reported and body mass index (BMI) in kg/m^2^ was estimated to classify body weight status [51]. BMI was calculated by dividing weight in kilograms over height squared in centimetres [51]. Previous papers stated that self-reported weight and height are acceptable for determining BMI [52].

Data on socioeconomic factors (age, education, income, race, and marital status) were collected to control for the influence of these confounding factors [53,54].

### 2.2. Statistical Analysis

IBM SPSS statistics version 25 (Chicago, IL, USA) was used for data analysis. The normality of the data was assessed by Shapiro–Wilk test. Descriptive data are presented as median and interquartile range (IQR) for data with non-normal distribution. Kruskal–Wallis test was used to compare continuous data among the aMD adherence categories (low, medium, high). Categorical data among the three aMD adherence categories were compared through Chi-squared test. The Bonferroni method was used to correct for multiplicity in data.

The normality of the food groups’ data was assessed, and appropriate transformation was undertaken when high skewness was detected in the data. Kaiser–Meyer–Olkin (KMO) measure of sampling adequacy and Bartlett’s test of sphericity were conducted to check the appropriateness of factor analysis. Results revealed a large KMO of 0.75 (>0.5) and a very significant Bartlett’s test (*p* < 0.00001) with an approximate Chi-square of 832 and 55 degrees of freedom; therefore, factor analysis was deemed appropriate to use [55]. Additionally, the sample size of the present study (*n* = 244) is acceptable for conducting factor analysis [56,57]. To derive the number of factors from the food groups’ data, a scree plot was generated showing the factors that have an eigenvalue >1. A varimax rotation was assigned to calculate factor loadings for each factor (dietary pattern) based on the assumption that factors were not correlated. Simple linear regression models carried out for each factor (dietary pattern) were revealed to investigate the association between that factor (dietary pattern) and the following variables separately: perceived stress, depression scores, BMI (kg/m^2^), PA (Mets) and socioeconomic measures. The predictors with significant association were then included in a multiple linear regression model of the diet pattern along with the other significant predictors. Some categories of the socioeconomic measures were merged together before inclusion in the regression models due to their small size (e.g., marital status (single/divorced/widowed, living together/married), parity (never, once/two times or more), religion (no religion, Christian, other), education (No qualification/Certificate of Secondary School (CSE)/General Certificate of Secondary School (GCSE), A-level/higher education), ethnicity (white, other), smoking status (smoker, non-smoker), income (below the average, above the average), parents occupation (employee, other)).

## 3. Results

A sample of 252 women participated in the study, and after screening eight were excluded since they did not meet the eligibility criteria (e.g., food intolerance/food allergy/chronic disease). In total, the data of 244 women were included in the analysis of the present study. Overall, participants had an average age of 24 years, were mainly of white ethnicity, single, non-smokers, and their parental educational level was mainly O-level or GCSE examinations taken at 16 years. In addition, 47% of the total sample had a moderate level of physical activity (Table 1 and Table 2).

The participants’ characteristics are reported across the three categories of the Alternate Mediterranean Diet Scores (aMDS: low, medium, and high). The majority of the 244 participants had a medium adherence to aMD (46%), followed by 39% having low adherence, and only 15% of participants had high adherence to aMD. 

There was a significant association between perceived stress and diet quality. Medium to high levels of stress were more likely to be reported by participants (73%) with a low adherence to aMDS (X^2^ (2, *n* = 244) = 14.08, *p* = 0.001). Pairwise comparisons showed that stress was different between low and high aMD adherence (*p* = 0.005) and between low and medium aMD adherence categories (*p* = 0.003) but not between medium and high adherence categories (*p* = 0.467). 

BMI was also found to be different among aMD adherence groups (X^2^ (4, *n* = 244) = 14.815, *p* = 0.005) (Table 1). Participants who had normal BMI were more likely to have high aMDS (72%) compared to those who were underweight (8%) and overweight/obese (17%). The physical activity level of participants differed across the three categories (X^2^ (4, *n* = 244) = 11.92, *p* = 0.018). A higher percentage of participants with high aMDS adherence were engaging in moderate (56%) and high (33%) physical activity levels whereas those with low aMDS adherence were engaging in low (41%) and moderate (41%) physical activity levels. 

Income per year showed a significant, but weak, association with adherence to aMDS (X^2^ (10, *n* = 244) = 18.48, *p* = 0.047). However, adherence to aMD was not associated with any other socio-demographic characteristics (Table 2).

### Factor Analysis

**Figure 1:**
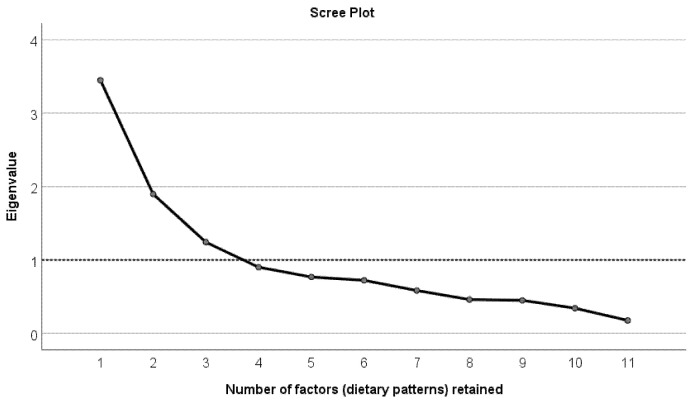
Scree plot showing the number of factors (dietary patterns) retained from factor analysis on the *X*-axis and the eigenvalues on *Y*-axis.

Figure 1 demonstrates a scree plot showing the number of factors (dietary patterns) retained from factor analysis. As shown in the scree plot, the number of factors (dietary patterns) with eigenvalue ≥1 is 3. The three factors (dietary patterns) explained 60% of the total variance in data. 

Table 3 demonstrates the 11 food groups with factor loadings for the three factors (dietary patterns). Coefficients with absolute value below 0.3 for each factor were suppressed, therefore five food groups were assigned to factor 1 (DP-1), three to factor 2 (DP-2), and three to factor 3 (DP-3). The first dietary pattern (DP-1) had high factor loadings for the following food groups: fats and oils, sugars and snacks, alcoholic beverages, cereals, and red and processed meat and was labelled “Western-style” dietary pattern. DP-2 had high factor loadings for food groups such as fish and seafood, eggs, and milk and milk products and was labelled “high-quality protein” dietary pattern. The third dietary pattern (DP-3) was labelled “vegetarian-like” dietary pattern with factor loadings high for fruits, vegetables, and nuts and seeds food groups.

Regression analysis that was used to examine the association between the three dietary patterns (DPs), which were derived from factor analysis, and all other variables indicated the following: In the first model, DP-1 was positively associated with stress (*p* = 0.005) and negatively with age (*p* = 0.004) and smoking (*p* = 0.005) (Table 4). DP-1 was common among young, smokers, and highly stressed women. Model 2 of the second dietary pattern showed that DP-2 was negatively associated with mother’s educational level (*p* = 0.019) (Table 4). The second dietary pattern (DP-2) was common among participants who had mothers with lower educational level. The third dietary pattern (DP-3) was common among normal weight people who had low stress level and non-white. As shown in Table 4, DP-3 was negatively associated with stress (*p* < 0.001), BMI (*p* = 0.001), and ethnicity (*p* = 0.013).

## 4. Discussion

This is the first study to investigate the association between perceived stress and diet quality/patterns among women of reproductive age in the UK. The association between stress and diet quality/patterns has recently gained the interest of health researchers, especially that diet is a main modifiable risk factor of obesity and many chronic diseases [21]. In the present study, diet quality/patterns analysis was used, rather than individual-nutrient assessment, because it allows the description of the whole diet of the population and is considered essential in understanding the relationship between dietary consumption and diet-related diseases [16]. Additionally, the association between stress and single nutrients is difficult to investigate since they are never consumed separately but rather within meals, and they metabolically interact with one another [16]. Our findings indicate that stress is associated with lower diet quality where 73% of participants who had low adherence to the alternate Mediterranean Diet (aMD) had a high stress level. Therefore, stress-coping strategies and programs for women of reproductive age should be implemented to prevent unhealthy eating habits and poor diet quality and their adverse health consequences.

Participants in this study were recruited from a university setting and included both students and employees (18–49 years old) to provide a more representative sample of reproductive aged women of this setting.

The a-priori assessment of diet quality indicated an overall medium adherence to the alternate Mediterranean Diet index (46% of the total sample). Similar results were found in the US where 43% of women of reproductive age (*n* = 248) had a moderate adherence to the Mediterranean Diet [58] and in the UK where most workplace females (*n* = 426) were moderate adherers to the Mediterranean Diet Index (*n* = 346) [59]. Similarly, our research team has previously assessed diet quality by the Mediterranean Diet Index, among 123 women of reproductive age in the UK and also reported an overall moderate adherence [60]; the alternate Mediterranean Diet Index was used in the present study because it has been considered more reflective of MD for non-Mediterranean countries such as the UK [61]. In this context, women of reproductive age should be supported with nutrition counselling and education, in addition to reproductive health care services, to further enhance their diet quality [62].

The a-posteriori dietary approach (Table 4) corroborated further the negative association between stress and healthy diet quality/patterns and offered additional dietary insight by highlighting the types of food groups that might contribute to this association. It showed that stress was positively associated with the Western-style dietary pattern (DP-1) consisting of fats and oils, sugar and snacks, alcoholic beverages, red/processed meat, and cereals (*p* = 0.005) and negatively with the vegetarian-like dietary pattern (DP-3) consisting of fruits, vegetables, nuts and seeds (*p* < 0.001). These findings agree with other studies targeting the association between stress and diet. For instance, El-Ansari et al. [16] assessed stress levels using the Perceived Stress Scale and nutritional habits through a 12-food item FFQ and found that among female university students in the UK, stress was significantly associated with poorer diet quality resembled by high intake of sugar, snacks, fat, and low intake of unsaturated fats, fruits, and vegetables. Additionally, Isasi et al. [25] found that stress was negatively linked with diet quality (Alternate-Healthy Eating Index 2010) among Hispanic/Latino females in the US. Similarly, Groesz et al. [63] targeted 561 females from the US and found that highly stressed females reported high consumption of unhealthy foods (fast food, sweets, etc.) and low consumption of whole grains, fruits, and vegetables as assessed via a food frequency questionnaire. In another study conducted among females across three countries (Germany, Poland, and Bulgaria), a positive association between stress and poor dietary patterns was reported [26]. Habhab et al. [64] also assessed the link between stress and food restraint and diet quality/patterns among 40 women of childbearing age via mixed-design analysis of variance and found that women with poorer diet quality had a high stress level. These findings were corroborated by our recent systematic review and meta-analysis [30] that was the first to examine the association between perceived stress and diet quality in women of reproductive age. The systematic literature review included 24 studies (8 had diet quality as the primary outcome and 16 assessed food frequency of consumption) with a total of 41,033 participants. Overall, the 16 studies on food intake and frequency of consumption (*n* = 33,477) found that stress was associated with high intake of fat, fast food, sweets, processed foods, and low intake of fruits, vegetables, whole grains, and legumes. The meta-analysis included the 8 studies on diet quality (*n* = 7556) and reported a significantly negative association between stress and diet quality (r  =  −0.35, *p* < 0.001, 95% CI (−0.56; −0.15)).

On the contrary, some studies reported different findings. For example, Richardson et al. [24] assessed stress through the 14-item PSS and diet quality via Healthy Eating Index-2010 among 101 childbearing aged women (aged 18–44 years) and found no association between stress and diet quality. Similarly, Ferranti et al. [65] found no association between perceived stress and diet quality among 433 females in the US who were university and health centre employees. The study assessed stress via the 14-item PSS and diet quality via a-priori approach using three diet quality indices: Alternate Healthy Eating Index, Mediterranean Diet Index, and Dietary Approach to Stop Hypertension Index. Two other studies in Egypt [66] and Iran [67] among women of childbearing age also found no significant association between diet quality and stress.

Discrepancies in findings between these studies and the present study might be explained by variations in sample sizes, diversity of the tools used to assess variables, and difference in the population from which the sample was taken. For instance, Richardson et al. [24] recruited 101 women and Widaman et al. [23] recruited 75 females. On the other hand, the present study recruited 244 participants. Secondly, most studies on the association between perceived stress and diet quality in women of reproductive age have used 24-h recalls as the dietary assessment tool [21,22,23,24,25] whereas the EPIC food frequency questionnaire, which measures a wide variety of food items and the frequency of consumption over the past one year, was used in the present study. 

In understanding the stress/diet relationship, studies have argued that the association between perceived stress and dietary quality/patterns is bidirectional: psychological stress symptoms could be associated with behaviours that are considered “health-compromising” that put the individual at risk of health problems [16]. For example, in a group of female students, high stress levels were associated with weight dissatisfaction and other health-compromising behaviours such as alcohol intake, binge eating and smoking, skipping breakfast [68]. Stressed people tend to consume high energy-dense foods to taper down their stressful emotions [69]. Adam et al. [69] suggested that the important reason behind these eating behaviours resulting from negative emotions and stress is the lack of eating control. This is when the consumption of high caloric and palatable foods relates to satisfaction and reward and becomes comfort eating during the stressful periods [69]. However, the absence of significant association between perceived stress and diet quality/patterns in some studies does not support these views. The findings of these studies can be explained by the following coping strategies that are not related to food such as spirituality that could attenuate the effect of stress on dietary behaviour [70]. Although these studies show no significant associations between perceived stress and diet quality, some environmental factors including stress coping strategies, cultural food traditions, cognitive factors (such as the knowledge of nutrition), and the cost of food might contribute to the dietary pattern and quality and must be further studied. Another explanation of the stress/diet relationship is derived from the fact that perceived stress causes physiological changes (in addition to psychological changes) to the human body that trigger food craving [71]. Upon stress, the hypothalamus and central nervous system secrete the hormone cortisol into the bloodstream which leads, if in high circulating concentrations, to the formation and accumulation of visceral fat in the body [69]. Additionally, several studies have pointed out that elevated levels of stress cortisol can be associated with increased food intake [72,73]. This is because perceived stress, and elevated serum cortisol, stimulate the secretion of the gastric hormone Ghrelin that increases appetite and food craving [71].

### Strengths and Limitations

The study has several strengths. To our knowledge, it is the first to assess the association between perceived stress and diet quality in women of reproductive age in the United Kingdom. Diet quality/patterns were assessed comprehensively through two approaches: the a-priori (hypothesis-driven) and the a-posteriori (data-driven) which gave robust results and clearer insight about the overall dietary quality/patterns of the study’s participants. Another strength of this study is the fact that the tools used to assess all variables were validated and standardised, such as the Perceived Stress Scale to assess stress levels [40], Becks Depression Inventory II to assess depression [42], in addition to the anthropometric and socioeconomic questions [74,75,76]. Furthermore, while most studies on the association between stress and diet utilised dietary recalls to assess dietary intake, this study used the EPIC food frequency questionnaire which is considered a gold standard dietary assessment tool [34,35].

On the other hand, there are several limitations which are worth acknowledging. The cross-sectional design of the study made it hard to draw and generalise a definitive conclusion about the association between perceived stress and diet quality/patterns among women of reproductive age. Additionally, the convenience sample that was selected from a population of a UK university setting and consequently might not be representative of the general population of women of reproductive age. Although all variables were measured using validated and standardised tools, they have been self-reported by participants, which might have caused inaccuracy in the results. For example, anthropometric measures would be better estimated using advanced and more accurate tools such as Dual-Energy X-ray Absorptiometry (DEXA) which measures the whole-body composition including weight, height, fat mass, and fat-free body mass [77]. Similarly, the Perceived Stress Scale, that was used to assess stress levels of participants, was self-reported and hence participants might not have accurately recalled the stressful situations that occurred over the past weeks. A more accurate measure of stress should be used in future studies such as blood or salivary cortisol [78]. Moreover, food intake biomarkers (such as urinary and blood samples), which objectively measure the nutritional intake of individuals, should complement the food frequency questionnaires and other self-reported measures of dietary intake [79].

## 5. Conclusions

In conclusion, the negative association between perceived stress and diet quality (in both a-priori and a-posteriori approaches) that was found among a sample of women of reproductive age in the present study is important and merits further investigation. The results of this study have implications for future interventions which should include not only dietary but also other behavioural aspects to support lifestyle changes among women of reproductive age. In other words, the interventions are complex; they are more than simply changing the diet alone. Women of reproductive age seem to eat depending on the level of stress and therefore dietary interventions need to take that into consideration when applying it. Future randomised controlled trials with accurate measures should be implemented to further confirm this negative association.

## Figures and Tables

**Table 1:** Physical and mental characteristics of participants (*n* = 244).

Participants’ Characteristics(N (%))	Total Sample	Alternate Mediterranean Diet Adherence Categories			*p*-Value
		Low aMDS (0–3)	Medium aMDS (4–6)	High aMDS (7–9)	
		95 (39)	113 (46)	36 (15)	
Physical and lifestyle characteristics					
Age (years) #	24.0 (21.0–32.0)	23.0 (21.0–29.0)	25.0 (21.5–32.0)	24.0 (20.3–35.0)	0.277
Age (years) *					0.09
18–24	124 (51)	54 (57)	51 (45)	19 (53)	
25–34	77 (32)	26 27)	44 (39)	7 (19)	
35–49	43 (17)	15 (16)	18 (16)	10 (28)	
BMI (kg/m^2^) #	23.7 (20.9–27.9)	26.1 (21.5–49.4)	23.7 (20.6–27.5)	21.9 (20.3–23.9)	0.093
BMI *					0.005
Underweight	14 (6)	4 (4)	7 (6)	3 (8)	
Normal Weight	120 (49)	38 (40)	56 (50)	26 (72)	
Overweight/obese	108 (44)	52 (56)	50 (44)	6 (17)	
Physical Activity (METs-h/wk) #	1429 (464.3–2824.5)	1159 (330.0–2615.0)	1440 (479.3–2886.3)	2380 (1325.5–3464.3)	0.336
Physical Activity level *					0.018
Low (<600 MET minutes/week)	76 (31)	39 (41)	33 (29)	4 (11)	
Moderate (>600 MET minutes/week)	114 (47)	39 (41)	55 (49)	20 (56)	
High (>3000 MET minutes/week)	54 (22)	17 (18)	25 (22)	12 (33)	
Mental Health Indicator					
Stress #	29 (22.0–33.0)	31 (26.0–34.0)	27 (22.0–27.0)	26.5 (18.0–31.8)	0.002
Stress *					0.001
Low-Medium	103 (42)	26 (27)	58 (51)	19 (53)	
Medium-High	141 (58)	69 (73)	55 (49)	17 (47)	
Depression #	5 (2.0–12.0)	5 (2.0–13.0)	5 (2.0–11.0)	5 (1.0–13.0)	0.926
Depression *					0.07
Minimal (0–13)	191 (78)	73 (77)	90 (80)	28 (78)	
Mild (14–19)	28 (11)	15 (16)	10 (9)	3 (8)	
Moderate (20–28)	12 (5)	4 (4)	8 (7)	0 (0)	
Severe (29–63)	13 (5)	3 (3)	5 (4)	5 (14)	

METs-h/wk: Metabolic equivalents of tasks-hours per week, BMI: body mass index., GCSE: General Certificate of Secondary Education, O-level: ordinary level. *p*-values were derived through a Chi-squared test of independence to display differences in physical activity, mental health indicators, and BMI of participants across the three Alternate Mediterranean diet (aMD) scores categories. The differences between median (IQR) of physical, mental health, and lifestyle characteristics were explored with Kruskal–Wallis test and post-hoc pairwise comparisons. * represents N (%). # represents median (IQR). aMDS: alternate Mediterranean Diet Score.

**Table 2:** Socio-demographic characteristics of participants (*n* = 244).

Participants’ Characteristics (N (%))	Total Sample N (%)	Alternate Mediterranean Diet Adherence Categories			*p*-Value
		Low aMDS (0–3)	Medium aMDS (4–6)	High aMDS (7–9)	
		95 (39)	113 (46)	36 (15)	
Father’s education					0.626
No qualifications	23 (9)	8 (8)	11 (10)	4 (11)	
Certificate of Secondary education (CSE) taken at 14–16 years at a lower level than GCSE	57 (23)	28 (29)	25 (22)	4 (11)	
			
O-level or GCSE examinations taken at 16 years	71 (29)	23 (24)	35 (31)	13 (36)	
A-level school examinations taken at 18 years	45 (18)	18 (19)	19 (17)	8 (22)	
Higher education	48 (20)	18 (19)	23 (20)	7 (19)	
Mother’s education					0.399
No qualifications	16 (7)	5 (5)	9 (8)	2 (6)	
Certificate of Secondary education (CSE) taken at 14–16 years at a lower level than GCSE	47 (19)	24 (25)	20 (18)	3 (8)	
			
O-level or GCSE examinations taken at 16 years	82 (34)	26 (27)	41 (36)	15 (42)	
A-level school examinations taken at 18 years	44 (18)	17 (18)	18 (16)	9 (25)	
Higher education	55 (23)	23 (24)	25 (22)	7 (19)	
Father’s occupation					0.424
Working as an employee	92 (38)	35 (37)	42 (37)	15 (42)	
On a government sponsored training scheme	5 (2)	4 (4)	1 (1)	0 (0)	
Self-employed or freelance	68 (28)	28 (29)	30 (27)	10 (28)	
Working paid or unpaid for your own or your family’s business	31 (13)	13 (14)	15 (13)	3 (8)	
			
Doing any other kind of paid work	9 (4)	6 (6)	1 (1)	2 (6)	
Retired (whether receiving a pension or not)	36 (15)	8 (8)	22 (19)	6 (17)	
Long-term sick or disabled	3 (1)	1 (1)	2 (2)	0 (0)	
Mother’s occupation					0.266
Working as an employee?	101 (41)	38 (40)	43 (38)	20 (56)	
On a government sponsored training scheme	9 (4)	4 (4)	5 (4)	0 (0)	
Self-employed or freelance	25 (10)	6 (6)	14 (12)	5 (14)	
Working paid or unpaid for your own or your family’s business	17 (7)	9 (9)	7 (6)	1 (3)	
	20 (8)	11 (12)	6 (5)	3 (8)	
Doing any other kind of paid work					
Retired (whether receiving a pension or not)	34 (14)	8 (8)	22 (19)	4 (11)	
Looking after home or family	27 (11)	14 (15)	11 (10)	2 (6)	
Long-term sick or disabled	11 (5)	5 (5)	5 (4)	1 (3)	
Income per year					0.047
<£13,000	119 (49)	43 (45)	54 (48)	22 (61)	
£13,000 to £33,800	99 (40)	45 (48)	47 (41)	7 (19)	
>£33,800	26 (11)	7 (7)	12 (11)	7 (19)	
Parents’ annual income					0.432
<£13,000	36 (15)	14 (15)	15 (13)	7 (19)	
£13,000 to £23,400	51 (21)	21 (22)	25 (22)	5 (14)	
>£23,400 to £33,800	69 (28)	31 (33)	33 (29)	5 (14)	
>£33,800 to £52,000	47 (19)	16 (17)	20 (18)	11 (31)	
>£52,000	41 (17)	13 (14)	20 (18)	8 (22)	
Marital Status					0.46
Single	176 (72)	67 (71)	84 (74)	25 (69)	
Married	43 (18)	16 (17)	18 (16)	9 (25)	
Divorced	17 (7)	9 (9)	8 (7)	0 (0)	
Separated but still legally married	6 (2)	3 (3)	2 (2)	1 (3)	
Widowed	2 (1)	0 (0)	1 (1)	1 (3)	
Smoking					0.47
Current Smoker	56 (23)	25 (26)	23 (20)	8 (22)	
Ex-smoker	27 (11)	10 (11)	11 (10)	6 (17)	
Never smoked	161 (66)	60 (63)	79 (70)	22 (61)	
Religion					0.437
No religion	104 (43)	36 (38)	53 (47)	15 (42)	
Christian	105 (43)	45 (47)	42 (37)	18 (50)	
Buddhist	7 (3)	2 (2)	5 (4)	0 (0)	
Hindu	9 (4)	5 (5)	3 (3)	1 (3)	
Jewish	(0)	(0)	(0)	(0)	
Muslim	19 (8)	7 (7)	10 (9)	2 (6)	
Sikh	(0)	(0)	(0)	(0)	
Ethnicity					0.231
Mixed/multiple ethnic groups	10 (4)	5 (5)	4 (4)	1 (3)	
White	177 (73)	61 (64)	82 (73)	34 (34)	
Asian/Asian British	35 (14)	18 (19)	16 (14)	1 (3)	
Black/African/Caribbean/Black British	15 (6)	7 (7)	8 (7)	0 (0)	
Other ethnic group	7 (3)	4 (4)	3 (3)	0 (0)	
Parity					0.229
Never	189 (77)	72 (76)	92 (81)	25 (69)	
Once	26 (11)	14 (15)	6 (5)	6 (17)	
Two times or more	29 (12)	9 (9)	15 (13)	5 (14)	

aMDS: alternate Mediterranean Diet Score. GCSE: General Certificate of Secondary Education, O-level: ordinary level. *p*-values were derived through a Chi-squared test of independence to display differences in socio-demographics of participants across the three alternate Mediterranean diet scores (aMDS) categories.

**Table 3:** Orthogonally rotated (varimax) factor loadings for the 3 factors (dietary patterns) of the 11 food groups (grams/day).

11 Food Groups Derived from the European Prospective into Cancer and Nutrition (EPIC) Food Frequency Questionnaire	Factors (Dietary Patterns)		
	1	2	3
Fats and Oils (grams/day)	0.838		
Sugars and Snacks (grams/day)	0.738		
Cereals (grams/day)	0.712		
Alcoholic beverages (grams/day)	0.665		
Red and processed meat (grams/day)	0.553		
Fish and Seafood (grams/day)		0.821	
Eggs (grams/day)		0.809	
Milk and milk products (grams/day)		0.518	
Fruits (grams/day)			0.750
Vegetables (grams/day)			0.747
Nuts and Seeds (grams/day)			0.619

**Table 4:** Multiple regression models showing the association between each a-posteriori-derived diet pattern and its predictor variables.

Model	Predictor	Coefficient Estimate	*p*-Value
1 (DP-1) “fats & oils, sugars & snacks, alcoholic beverages, red and processed meat, and cereals” DP	Intercept	0.419	<0.001
	Stress	0.003	0.005
	Physical activity (METs-h/wk)	−0.0000002	0.395
	BMI	0.002	0.062
	Age	−0.003	0.004
	Father’s educational level (A-level/higher)	−0.027	0.107
	Mother’s educational level (A-level/higher)	−0.006	0.713
	Ethnicity (white)	0.026	0.128
	Father’s occupation (other)	0.015	0.369
	Mother’s occupation (other)	0.022	0.174
	Smoking status (smoker)	−0.05	0.005
	Participant’s income (above average)	0.026	0.098
2 (DP-2) “fish & seafood, eggs, and milk & milk products” DP	Intercept	0.441	<0.0001
	Stress	−0.002	0.14
	Depression	0.0001	0.676
	Mother’s education (A-level/higher)	−0.038	0.019
	Father’s occupation (other)	0.035	0.057
	Mother’s occupation (other)	0.018	0.313
	Participant’s income (above average)	0.033	0.069
3 (DP-3) “fruits, vegetables, and nuts & seeds” DP	Intercept	0.653	<0.001
	Stress	−0.005	<0.001
	Physical activity (METs-h/wk)	0.0000006	0.115
	BMI	−0.005	0.001
	Ethnicity (white)	−0.047	0.013
	Parent’s income (above average)	0.023	0.184
	Smoking (smoker)	0.033	0.092

DP: dietary pattern. METs-h/wk: metabolic equivalents of tasks-hours per week. Model 1 of the first dietary pattern was based on the following formula: DP − 1 = β0 + β1 Stress + β2 Physical activity + β3 BMI + β4 Age + β5 Father’s educational level + β6 Mother’s educational level + β7 Ethnicity + β8 Father’s occupation + β9 Mother’s occupation + β10 Smoking status + β11 Participant’s income. Model 2 of the second dietary pattern was based on the following formula: DP − 2 = β0 + β1 Stress + β2 Depression + β3 Mother’s education + β4 Father’s occupation + β5 Mother’s occupation + β6 Participant’s income. Model 3 of the third dietary pattern was based on the following formula: DP − 3 = β0 + β1 Stress + β2 Physical activity + β3 BMI + β4 Ethnicity + β5 Parent’s income + β6 Smoking.
